# Mastering your fellowship

**DOI:** 10.4102/safp.v63i1.5364

**Published:** 2021-08-30

**Authors:** Klaus B. von Pressentin, Mergan Naidoo, Idongesit S. Ukpe, Tasleem Ras

**Affiliations:** 1 Division of Family Medicine, School of Public Health and Family Medicine, Faculty of Health Sciences, University of Cape Town, Cape Town, South Africa; 2 Department of Family Medicine, University of KwaZulu-Natal, Durban, South Africa; 3 Department of Family Medicine, University of Pretoria, Pretoria, South Africa

**Keywords:** family physicians, FCFP (SA) examination, family medicine registrars, postgraduate training, national exit examination

## Abstract

The series, ‘Mastering your Fellowship’, provides examples of the question format encountered in the written and clinical examinations, Part A of the FCFP (SA) (Fellowship of the College of Family Physicians [SA]) examination. The series is aimed at helping family medicine registrars (and their supervisors) prepare for this examination. Model answers are available online.

This section in the South African Family Practice journal is aimed at helping registrars prepare for the Fellowship of the College of Family Physicians (FCFP [SA]) Final Part A examination (Fellowship of the College of Family Physicians). It will provide examples of the question formats encountered in the written examination such as multiple choice question (MCQ) in the form of single best answer (SBA – Type A) and/or extended matching question (EMQ – Type R); short answer question (SAQ), questions based on the Critical Reading of a journal article (evidence-based medicine) and an example of an objectively structured clinical examination (OSCE) question. Each of these question types are presented based on the College of Family Physicians blueprint and the key learning outcomes of the FCFP programme. The MCQs will be based on the 10 clinical domains of family medicine, the SAQs will be aligned with the five national unit standards and the critical reading section will include evidence-based medicine and primary care research methods.

This edition is based on unit standard 1 (critically reviewing new evidence and applying the evidence in practice), unit standard 2 (evaluate and manage a patient according to the bio-psycho-social approach), unit standard 3 (facilitate the health and quality of life of the family and community), and unit standard 4 (facilitate the learning of others regarding the discipline of family medicine, primary health care (PHC), and other health-related matters). The theme for this edition is orthopaedics. We suggest that you attempt answering the questions (by yourself or with peers/supervisors), before finding the model answers online: http://www.safpj.co.za/.

Please visit the Colleges of Medicine website for guidelines on the Fellowship examination: https://www.cmsa.co.za/view_exam.aspx?QualificationID=9.

We are keen to hear about how this series is assisting registrars and their supervisors in preparing for the FCFP (SA) examination. Please email your feedback and suggestions to the corresponding author (K.B.v.P.).

## Extended Matching Question

### Theme: Management of backache

1. A 40-year-old male presents with lower backache for 3 months and reports no radiation. He works as an administration clerk at a large warehouse. He is 1.7 m tall and weighs 85 kg. His vital signs are all normal. He has mild limitation in movement on forward flexion of his lumbar spine, his straight leg raise test is negative and he has no focal neurology. He reports that he has already tried paracetamol and ibuprofen and got only temporary relief.
2. A 70-year-old female presents with backache for the last 4 weeks. She is a pensioner and states that the pain interferes with her gardening. She is 1.65 m tall and weighs 56 kg. Her vital signs and general physical examination are normal. She has mild limitation in movement on forward flexion of his lumbar spine, her straight leg raise test is negative and she has no focal neurology. She reports that she has already tried paracetamol and ibuprofen for the last 3 weeks with minimal relief.

For each scenario described above, choose the most appropriate next step in the management of this patient, from the list of options below. Each option may be used once, more than once, or not at all.

- Amitriptyline
- Bed rest
- Blood tests
- Exercise and posture modification
- Psychotherapy
- Refer to the orthopaedic surgeon
- Spinal manipulation
- Traction
- X-rays of the spine

*Short answer:* 1 D, 2 I

### Background information to clarifying the underlying reasoning behind the short answers

Lower back pain (LBP) is a common symptom in primary care and clinical practice guidelines evolve with time as new evidence is thrown into the ‘meta-analysis mixer’. However, basic principles of evaluation form the cornerstone in the management of LBP. The history and examination are critically important in excluding red flags which include the following:

- Malignancy: A history of a diagnosed malignancy or specific alarming symptoms (local symptoms such as pain at night/bone pain, or constitutional symptoms such as unexpected weight loss) may point in this direction.
- Infection: Fever and comorbid human immunodeficiency virus (HIV) infection may be pointers in this direction.
- Fracture: A history of trauma, prolonged steroid use and the elderly are suggestive clues.
- Spinal pathology: Clinical examination yielding local neurological signs are highly suggestive.
- Age: under 18 years of age or over 50 years of age and new onset LBP.

Evaluation of the LBP should also consider the following yellow flags:

- Beliefs that pain and activity are harmful.
- Treatment preferences that do not fit with the best practice.
- Lack of social support.

Examination of the back uses the ‘look, feel, move’ approach:

Look:

- At the patient’s gait
- For spinal deformity or swelling
- For muscle wasting
- For skin changes

Feel:

- For deformities between spines, paraspinal muscles, sacroileac joints
- The abdomen
- Tenderness (vertebral/paravertebral)

Move:

- Flexion. Extension, lateral extension, and rotation of the cervical and lumbar spine

Special tests:

- Full lower limb neurological examination
- Straight leg raising test
- Slump test
- Schober’s test

**FIGURE 1:**
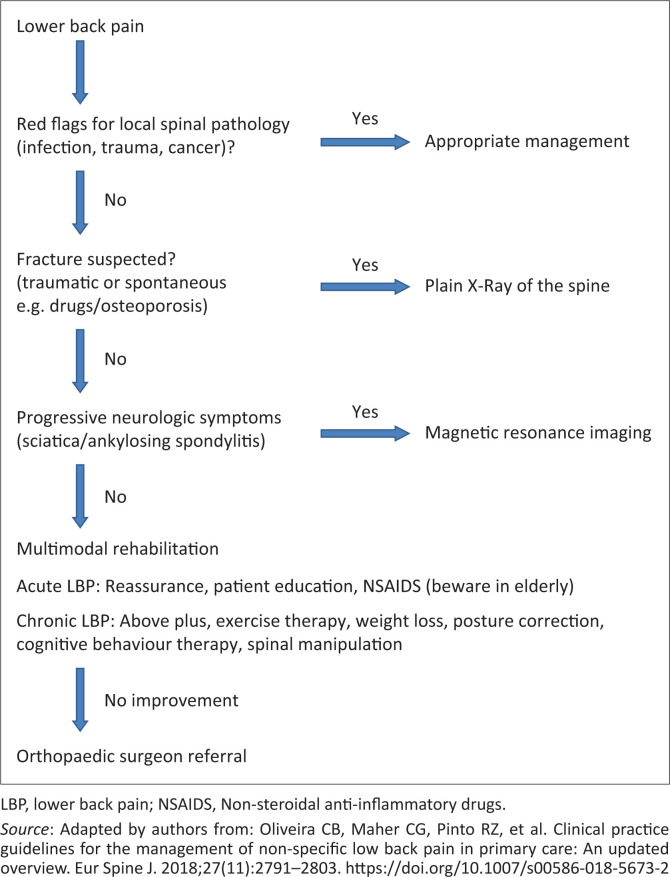
Suggested algorithm for managing low back pain.

The evaluation of the backache can follow the flowchart in Figure 1.

Practice guidelines around the world vary, but most programmes recommend a multimodal approach to spinal rehabilitation. This includes patient education, pharmacotherapy, weight loss, posture modification, exercise therapy to strengthen core muscles and improve strength and flexibility, cognitive behaviour therapy and spinal manipulation therapy (SMT). Spinal manipulation therapy has shown some promise when evaluated in several metanalyses but must be tailored to suit the specific need of the patient. Other physiotherapy modalities evaluated have not been met with any firm evidence-based recommendations.

### Back to the extended matching questions

The first patient is overweight, has no red flags and his occupation places him at risk of poor posture and poor physical conditioning. Hence, the recommended next step after patient education would be postural modification and exercise prescription. The second patient is an elderly, post-menopausal woman. She is at risk of osteoporosis. So one needs to exclude a spinal fracture; hence the need for a plain X-ray.

### Further reading

- Oliveira CB, Maher CG, Pinto RZ, et al. Clinical practice guidelines for the management of non-specific low back pain in primary care: An updated overview. Eur Spine J. 2018;27(11):2791–2803. https://doi.org/10.1007/s00586-018-5673-2
- Last AR, Hulbert K. Chronic low back pain: Evaluation and management. Am Fam Physician. 2009;79(12):1067–1074.
- Herndon CM, Zoberi KS, Gardner BJ. Common questions about chronic low back pain. Am Fam Physician. 2015;91(10):708–714.
- Mash B. How to examine and assess low back pain. In: Mash B, Blitz J, editors. SA family practice manual. 3rd ed. Cape Town: Van Schaik; 2015, p. 347–350.

## Short answer question: The family physician’s role as care provider and consultant

The South African Social Security Agency (SASSA) medical officer (MO) who assesses Disability Grant (DG) applicants at the district hospital where you work as the consultant raises a concern regarding hand disabilities resulting from injuries from fireworks that were managed at the hospital. The SASSA MO requests you to address the management of such injuries at the hospital to prevent or minimise disabilities.

(Total: 20 marks)

1. What would be your immediate response to the SASSA medical officer? (2 marks)
2. As a clinician and consultant family physician at the hospital, what actions would you personally take to try to address the management of fireworks hand injuries at the hospital? (12 marks)
3. On further discussion with the SASSA medical officer, how could you address this problem in the community? (4 marks)
4. As a specialist, how would you utilise the results of the actions you took to address the management of hand injuries at the hospital? (2 marks)

### Model answers

1. **What would be your immediate response to the SASSA medical officer? (2 marks)**

- Thank the SASSA MO for the concern and the request. Offer to assist the SASSA MO to have the concern raised with the hospital clinical manager/management.
- Thank the MO for highlighting the problem.
- I would fulfil my role as a leader of clinical governance responsible for ensuring quality of clinical care, with roles including ensuring clinical competence of staff and capacity building. I would serve as a bridge between the clinicians and management of the hospital, ensuring teamwork to resolve problems and building an enabling working environment and organisational culture.
- I would request more specific information from the MO on gaps in the clinical management noted – inadequate history/examination/investigations/management and rehabilitation?
- I would also want to know how big the problem is. How many such hand injuries has he seen? Over what period? What were the extend of the injuries?

2. **As a clinician and consultant family physician at the hospital, what actions would you personally take to try to address the management of fireworks hand injuries at the hospital? (12 marks)**

The consultant family physician is expected to be able to take clinical governance actions to try to address and improve the management of fireworks hand injuries at the hospital such as follows:

- Review of current literature and evidence-based management of firework related hand injuries and their management *(see further reading suggestions below)*.
- Carrying out an audit of fireworks hand injuries seen and managed at the district hospital.
- Conducting morbidity and mortality meetings to review cases where management was inappropriate and potentially landed up in complaints/claims/litigation.
- Carrying out quality improvement cycles to address structure/process/clinical outcome issues in the hospital/skills availability audit for the management of fireworks hand injuries at the district hospital.
- Capacity building in collaboration with the referral hospital orthopaedic surgeon to organise a multidisciplinary continuing professional development (CPD) meeting on the management of fireworks hand injuries at the district hospital. This CPD meeting should include the multidisciplinary team, including nursing, occupational therapy and physiotherapy. It may also include more practical sessions on examination of hands, acute management (correct casts), referral pathways, and rehabilitation.
- Assessing new staff for clinical competence in management of hand injuries.
- Development of protocols, in collaboration with the referral hospital orthopaedic surgeon, for the management of fireworks hand injuries at the district hospital.

3. **On further discussion with the SASSA medical officer, how could you address this problem in the community? (4 marks)**

A family physician is expected to be versed in community oriented primary care (COPC) and be able to address health problems like fireworks hand injuries at the community level in a collaborative manner such as teaming-up with the SASSA MO to initiate a community education programme on fireworks safety in the district. The COPC approach could include the following steps:

- Approach important role players in the community (ward counsellor, teachers, church).
- Form a team including important role players to address priority issues in the community (you would raise firework related injuries as a problem highlighted from the hospital).
- Do an institutional analysis of available resources in the community to assist those with permanent hand injuries/education.
- Implementation of ideas from the team (community education).
- Monitoring and reassessment/evaluation.

4. **As a specialist, how would you utilise the results of the actions you took to address the management of hand injuries at the hospital? (2 marks)**

As a specialist, the consultant family physician is expected to be able to share experiences with the wider scientific community by the following ways:

- Presentation of the audit findings and quality improvement initiatives to the local and district management teams.
- Engagement with local community leaders and community newspapers and media to highlight the problem of injuries caused by fireworks.
- Conference presentations and publications in scientific journals (providing that ethical approval has been obtained).

*Further reading:*

- Pilling T, Govender P. Profile and management of the firework-injured hand. S Afr Fam Pract. 2016;58(2):48–53. https://doi.org/10.1080/20786190.2015.1125167
- Connell L. A clinical governance handbook for District Clinical Specialist Teams [homepage on the Internet]. Durban: Health Systems Trust; 2014 [2021 Jul 14]. Available from: https://www.hst.org.za/publications/HST%20Publications/Clinical%20Gov%20Handbook_LR_24Oct2014.pdf
- Marcus TS. COPC – A practical guide [homepage on the Internet]. Pretoria: Department of Family Medicine, University of Pretoria; September 2018 [2021 Jul 14]. Available from: https://www.researchgate.net/publication/327495860_COPC-_A_Practical_Guide

## Critical appraisal of quantitative research

Read the accompanying article carefully and then answer the following questions (total 40 marks). As far as possible use your own words. Be guided by the allocation of marks with respect to the length of your responses.

- Esaadi M, Paruk F, Cassim B. Prevalence and clinical risk factors for morphometric vertebral fractures in older subjects in KwaZulu-Natal. J Endocrinol Metab Diabetes S Afr [serial online]. 2021 [cited 2021 Jul 14];26(1):29–33. Available from: http://www.jemdsa.co.za/index.php/JEMDSA/article/view/763
  1. What research question did the authors attempt to answer in this study? Comment on whether this was a clearly focused question with reference to the PICO framework (Population of interest, Intervention or Issue of Interest, Comparison intervention of interest, primary Outcome of interest). (5 marks)
  2. Considering the introduction section in this article, identify three sentences/phrases that best reflect the authors’ starting point, from which the rationale for the research is further explained and/or elaborated on (more than one correct answer possible). (3 marks)
  3. Comment critically on the sample used in this study. Were the groups (cases and controls) comparable and were these groups matched appropriately? Comment critically on the authors’ choice of performing a secondary analysis of an existing dataset from a primary study. (6 marks)
  4. Comment critically on whether objective and validated measurement methods were used to measure the exposure and outcomes? (6 marks)
  5. Were confounding factors identified and were strategies to deal with confounding factors stated? (4 marks)
  6. ‘Subjects with a VF (vertebral fractures) had a significantly lower median BMD (bone mineral density) at spine compared with subjects without a VF (0.745 g/cm^2^ [IQR 0.639–0.958 g/cm^2^] vs. 0.870 g/cm^2^ [0.722–0.988 g/cm^2^], *p* = 0.020)’. Please explain what you understand by this statement in relation to the Odds Ratio/Hazard Ratio of 0.137 and the associated 95% confidence interval in Table 4 [*Esaadi et al. 2021*]. Also define IQR (interquartile range). (6 marks)
  7. Using the acronym READER (Relevance, Education, Applicability, Discrimination, Evaluation and Reaction) try to analyse this article’s applicability to your own context (take home message). (10 marks)

*(Total: 40 marks)*

### Model answers to questions

**Model answers:**

1. **What research question did the authors attempt to answer in this study? Comment on whether this was a clearly focused question with reference to the PICO framework (Population of interest, Intervention or Issue of Interest, Comparison intervention of interest, primary Outcome of interest). (5 marks)**

In the text, the authors state that the aim of the study is to further define the prevalence and risk factors for vertebral factors (VFs) in women and men in South Africa – there is no mention of age or limitation to a few facilities in KwaZulu-Natal. However, in the abstract they state that the study was undertaken to compare the demographic profile, clinical risk factors and bone mineral density (BMD) in subjects aged 60 years and over with and without morphometric VFs. The term ‘morphometric VFs’ was not clearly defined – the only explanation was with reference to the radiological assessment, namely when ‘thoracic and lumbar vertebrae were deemed abnormal’.

The PICO framework (*P*atient group, *P*atient Problem or *P*opulation of interest, *I*ntervention or *I*ssue of *I*nterest, *C*omparison intervention of interest, primary *O*utcome of interest) is generally used to help frame or focus the research question. The framework may be tailored to the research question type (treatment, prevention, diagnosis, prognosis, or aetiology) or study design (quantitative compared to qualitative).

Using the PICO framework for this study, the population of interest (*P*) would be adult patients (women and men) aged 60-years and older; the issue of interest (*I*): the presence of morphometric VFs and its associated risk factors. Although there is no explicit comparison intervention of interest, the context (*C*) is that of five public sector regional hospitals in the eThekwini area, KwaZulu-Natal, which provide an orthopaedic service. The outcome of interest (*O*) would be the presence of morphometric VFs and its associated risk factors, especially with regard to demographic profile, clinical risk factors and BMD.

This study therefore aimed to answer a specific question. At this stage of the critical appraisal process, it remains to be seen if this focused research question would be able to provide sufficient evidence to further define the prevalence and risk factors for VFs in women and men.

2. **Considering the introduction section in this article, identify three sentences/phrases that best reflect the authors’ starting point, from which the rationale for the research is further explained and/or elaborated on (more than one correct answer possible). (3 marks)**

Potential options include:

- Vertebral fractures (VFs) are the most common complication of osteoporosis.
- In a multinational study in postmenopausal women newly diagnosed with osteoporosis, 68% of the subjects had an undiagnosed VF.
- These fractures often develop insidiously over time, and at presentation patients may have multiple prevalent fractures, with progressive loss of stature and disability.
- Due to their silent nature, most fractures are undiagnosed and not referred for appropriate treatment.
- Whilst the prevalence and clinical risk factors for VFs are established in developed countries, there are limited studies from developing countries.
- South Africa has a unique multi-ethnic population, in whom risk factors and disease profile may vary significantly.
- Recent studies question the notion that VFs are rare in black women.
- There is no information, however, on Indian women in SA nor are there any data on men.

3. **Comment critically on the sample used in this study. Were the groups (cases and controls) comparable and were these groups matched appropriately? Comment critically on the authors’ choice of performing a secondary analysis of an existing dataset from a primary study. (6 marks)**

The study population comprised 197/200 of the control subjects enrolled in a primary study who had vertebral radiographs. The initial study was conducted in five public sector regional hospitals in the eThekwini area, KwaZulu-Natal, which provide an orthopaedic service. For this subsequent analysis presented in this article, the cases are those healthy volunteers from the initial study who have incidentally diagnosed, missed VFs; and the controls for the subsequent analysis are those health volunteers without VFs.

These control subjects were volunteers who were able to give informed consent and did not have a prior history of osteoporosis or hip fractures. However, osteoporosis is often only diagnosed when someone has a fracture. So, a prior history does not exclude the diagnosis of osteoporosis.

In the methods section no details are given on the sample size that is needed to achieve the power necessary to reach conclusions which can be extrapolated to the study population. It is not clear whether a sample size of 197 is sufficiently powered.

Sources of bias could therefore include sampling bias, namely the non-probability sampling technique to sample the controls for the primary longitudinal study undermines the external validity (the ability of the study’s results to be generalised to the entire population). As they wanted to establish the prevalence, they could have used a cross-sectional study rather than trying to match those with established osteoporosis with those who may not have osteoporosis.

Table 1 in this article demonstrates that the cases and control groups are not well matched, especially as this analysis was not part of the original/primary study design. The cases (patients with VFs) were only 41, compared to 156 control subjects. The case subjects were significantly older (*p* = 0.009).

Therefore, this article describes an analysis of historical data from a previous study which aimed to match healthy volunteers as controls to patients with osteoporotic hip fractures (cases). Interestingly, no reference is provided to the previous primary longitudinal study on osteoporotic hip fractures, from which the historical dataset is drawn.

4. **Comment critically on whether objective and validated measurement methods were used to measure the exposure and outcomes? (6 marks)**

For a research tool or tool to be used in a study, it must be validated for the study setting. The authors used the same criteria for identification of cases and controls, namely the diagnosis of VFs using radiological assessment. Antero-posterior (AP) and lateral radiographic views of the thoracic and lumbar spine were acquired using a standardised protocol. All radiographs were reported by a single blinded experienced specialist radiologist. Thoracic and lumbar vertebrae were deemed abnormal (morphometric fracture), using the ‘semi-quantitative Genant method’.

The methods to determine the presence of risk factors included a structured questionnaire which was used to collect demographic details, education level, clinical risk factors for osteoporosis and gynaecological history. The authors did not state whether the Danish Health and Morbidity Survey (to assess alcohol use) and the World Health Organization (WHO) Monitoring Trends and Determinants in Cardiovascular disease scale (MONICA scale) (to determine smoking exposure), were validated for the South African context. Similarly, it is not clear whether the International Osteoporosis Foundation (IOF) calcium intake diary (to quantify calcium intake) was valid for the local context. The authors did state that functional level was assessed using the validated Physical Self-Maintenance Scale (PSMS) and the Lawton Instrumental Activities of Daily Living scale (IADL) scales, which have ‘good inter-rater reliability at 0.87 and 0.91, in multiple studies, respectively’. It is not clear from these references (20 and 21), whether these reliability tests were performed in the South African or similar context.

It is not clear how the structured questionnaire was administrated (self vs. researcher). From the results section, we learn that around 40% of respondents had no schooling or had only primary level schooling. Usually, the education level of the survey population should be considered when thinking about how easy it will be for the respondents to interpret and answer a question. Ordinary and everyday language is preferred. Participants with poor English language ability may have required support or an interpreter.

Weight and height were recorded, and body mass index (BMI) was calculated. Presumable these measurements were obtained at the five hospitals, and it is not clear if the researchers ensured calibration and consistency in these measurements.

Fortunately, the BMD measurements at the hip and spine were performed in a standardised manner. To ensure reliability a spine phantom was scanned weekly to determine the coefficient of variation, which was < 1.5%. Bone mineral density T-scores were categorised according to the WHO diagnostic criteria.

5. **Were confounding factors identified and were strategies to deal with confounding factors stated? (4 marks)**

Confounding is often referred to as:

[*A*] ‘mixing of effects’, wherein the effects of the exposure under study on a given outcome are mixed in with the effects of an additional factor (or set of factors) resulting in a distortion of the true relationship (Esaadi et al. 2021).

The outcome of interest is the presence of morphometric VFs. The authors used the available literature to identify and collect data from the subjects on potential risk factors, especially with regard to demographic profile (age, gender, ethnicity, education level), clinical risk factors (BMI, smoking history, alcohol intake, sun exposure, dietary calcium intake, paternal and prior history of fractures, functional level) and BMD. These factors are presented in Table 1 (Esaadi et al. 2021). Table 3 (Esaadi et al. 2021) also compares the gynaecological history between cases and controls, and Table 4 (Esaadi et al. 2021) compares BMD between cases and controls. The authors seem to have considered all the common factors associated with the outcome of interest, osteoporotic VFs. Morphometric VFs are associated with osteoporosis, which may also be caused by rare causes such as other primary and secondary endocrine conditions, such as growth hormone, parathyroid and renal conditions.

The potential for confounding should be considered in the design, implementation and analysis stages of the study. Factors which might be associated with the outcome need to be measured. Multivariate statistical analysis allows for adjustment of multiple variables simultaneously via mathematical modelling, can also be used to ‘control’ for confounding. In this study, inferential statistics were applied to compare cases and controls.

6. **‘Subjects with a VF (vertebral fractures) had a significantly lower median BMD (bone mineral density) at spine compared with subjects without a VF (0.745 g/cm** ^**2**^ **[IQR 0.639–0.958 g/cm** ^**2**^ **] vs. 0.870 g/cm** ^**2**^ **[0.722–0.988 g/cm** ^**2**^ **], *p* = 0.020)’. Please explain what you understand by this statement in relation to the Odds Ratio/Hazard Ratio of 0.137 and the associated 95% Confidence Interval in Table 4 [*Esaadi et al. 2021*] of the article. Also define IQR (interquartile range). (6 marks)**

The odds ratio (OR) is the ‘measure of association’ for a case-control study. It quantifies the relationship between an exposure (such as eating a food or attending an event) and a disease in a case-control study. The OR is calculated using the number of case-patients who did or did not have exposure to a factor (such as a particular food) and the number of controls who did or did not have the exposure. The OR tells us how much higher the odds of exposure are amongst case-patients than amongst controls.

Conversely, the hazard ratio (HR) is a comparison between the probability of events in a treatment/case group, compared to the probability of events in a control group. The HR has also been defined as the ratio of (risk of outcome in one group)/(risk of outcome in another group), occurring at a given interval of time (typically used to compare time to event data between two treatment groups). A HR of 1 means that both groups (case and control) are experiencing an equal number of events at any point in time.

In this study, the authors aimed to describe the risk factors associated with the presence of VFs in the cases subjects. Confusingly, the authors used the combined ‘OR/HR’ term in the inferential statical results table (Table 4 of Esaadi et al. 2021). It would have been helpful to the reader to use either the OR or HR in the table and/or text, to guide the interpretation. The median BMD at spine was significantly lower in case subjects with VFs (a median of 0.75 g/cm^2^ in cases, compared to a median of 0.87 g/cm^2^ in controls). An OR of 0.137 should therefore imply that the odds of having a lower BMD at the level of the spine were lower in cases (subjects who were experiencing the event of interest, VFs) compared to the control group, which is a false interpretation, as a lower BMD should be resulting in more VFs. Logically speaking, it would make more sense to interpret this result as an HR instead; the HR of having a VF was 0.137, reflecting the reduced risk of having a VF associated with a low BMD in the control group.

The Mann–Whitney U-test was used to compare differences between the two independent groups (it is used when the dependent variable is either ordinal or continuous, but the data is not normally distributed or non-parametric). Because only a sample of the population can be measured, confidence intervals (CIs) (precision) give a range in which you think the real answer lies with a given degree of certainty. For the OR/HR in relation to BMD and screening positive for VFs, we can be 95% certain that the CI of 0.02 – 0.95 contains the true population parameter for the OR/HR. In general, the larger the sample size, the smaller the CI, and vice versa. When the CI of a ratio crosses 1, that is, the range encompasses values showing increased and decreased risk, the statistical significance of the given ratio is weakened.

In descriptive statistics, the interquartile range (IQR), also called the midspread or middle 50%, is a measure of statistical dispersion, being equal to the difference between 75th and 25th percentiles, or between upper and lower quartiles. The IQR is used to describe the statistical dispersion of a non-parametric variable.

7. **Using the acronym READER (Relevance, Education, Applicability, Discrimination, Evaluation and Reaction) to analyse this article’s applicability to your own context (take home message). (10 marks)**

The READER format may be used to answer this question:

- Relevance to family medicine and primary care?
- Education – does it challenge existing knowledge or thinking?
- Applicability – are the results applicable to my practice?
- Discrimination – is the study scientifically valid enough?
- Evaluation – given the above, how would I score or evaluate the usefulness of this study to my practice?
- Reaction – what will I do with the study findings?

*The answer may be a subjective response but should be one that demonstrates a reflection on the change possible changes within the student’s practice within the South African public healthcare system. It is acceptable for the student to suggest how his or her practice might change, within other scenarios after graduation (e.g. private general practice). The reflection on whether all important outcomes were considered is therefore dependant on the reader’s own perspective (is there other information you would have liked to see?).*

*A model answer could be written from the perspective of the family physician employed in the district health system:*

This study is relevant to the African primary care context, as screening, diagnosing and treating osteoporotic VFs in a comprehensive manner represent core aspects of a high-quality, team-based PHC approach. The authors (Esaadi et al. 2021) stated that:

VFs usually … develop insidiously over time, and at presentation patients may have multiple prevalent fractures, with progressive loss of stature and disability. Due to their silent nature, most fractures are undiagnosed and not referred for appropriate treatment.

The authors did not describe the appropriate treatment options available in the public health sector, but this approach supports the preventative and promotive aspects of comprehensive PHC.

In terms of discrimination, the methodological concerns raised (sampling, validity of some of the questionnaires for the South African context and sample size) make any conclusions drawn questionable. Several of the findings in the study were not statistically significant because of the small sample size, and the sample was skewed towards Indian and African individuals. The study setting was also confined to public sector regional hospitals with orthopaedic services in an urban South African city. It would therefore not be possible to generalise the study findings to the wider South African setting, especially the primary care or district health services.

The authors motivated for an increased awareness and screening for osteoporosis in all South Africans, regardless of ethnicity. The authors recommended further studies to determine the population-based prevalence and clinical risk factors of VFs in SA to guide screening and management protocols. The study may be discussed with the local clinical team and used as a basis for creating awareness regarding the undiagnosed and potentially unmet need of osteoporosis in PHC. It is unlikely that this study will affect a change in policy direction, largely because of its limitations, but it could help make the case for further research of a more robust design. The individual primary care provider and his or her team may be reminded about the potential risk factors for osteoporosis, specifically in patients who present with known risk factors. Access to BMD measurements and osteoporosis-related medical therapy at PHC level is a significant constraint at primary care level. This will necessitate careful planning and care coordination in consultation with the specialist colleagues at the referral hospital.

### Further reading:

- Mash B, Ogunbanjo GA. African primary care research: Quantitative analysis and presentation of results. Afr J Prim Health Care Fam Med. 2014;6(1):1–5. https://doi.org/10.4102/phcfm.v6i1.646
- Skelly AC, Dettori JR, Brodt ED. Assessing bias: The importance of considering confounding. Evid Based Spine Care J. 2012;3(1):9. https://doi.org/10.1055/s-0031-1298595
- Pather M. Evidence-based family medicine. In: Mash B, editor. Handbook of family medicine. 4th ed. Cape Town: Oxford University Press, 2017; p. 430–453.
- Sedgwick P, Joekes K. Interpreting hazard ratios. BMJ. 2015;351:h4631. https://doi.org/10.1136/bmj.h4631
- Stevens R. Statistics in primary care research. In: Goodyear-Smith F, Mash B, editor. How to do primary care research. 1st ed. Boca Raton, FL: CRC Press, 2019; p. 161–165.
- Joannabriggs.org. Critical appraisal tools – JBI [homepage on the Internet]. 2021 [cited 2021 Jun 21]. Available from: https://jbi.global/critical-appraisal-tools
- The Critical Appraisals Skills Programme (CASP). CASP checklists [homepage on the Internet]. 2021 [cited 2021 Jun 21]. Available at: https://casp-uk.net/casp-tools-checklists/
- MacAuley D. READER: An acronym to aid critical reading by general practitioners. Br J Gen Pract. 1994;44(379):83–85.

## Objectively structured clinical examination scenario

### Objective of station:

This station tests the following:

- Ability to gather information, synthesise an assessment, and develop a management plan for a patient with chronic musculoskeletal pain.
- Application of relevant knowledge in the diagnosis and management of lumbar radiculopathy.

Type of station: Simulated consultation

### Equipment list:

- Middle aged role player
- Clinical examination findings
- X-ray image

### Instructions to candidate:

You are working in the outpatient department of the community health centre, and consult with the following patient, referred to you by the nurse practitioner.

Your task:

- Conduct a consultation with this patient, developing a comprehensive assessment and management plan.
- Write down your assessment and management plan.

### Instructions for the examiner

This station tests:

- Ability to gather information, synthesise an assessment, and develop a management plan for a patient with chronic musculoskeletal pain.
- Application of relevant knowledge in the diagnosis and management of lumbar radiculopathy.

This is an integrated consultation station in which the candidate has 15 min.

Familiarise yourself with the Assessor guidelines which details the required responses expected from the candidate.

**FIGURE 2:**
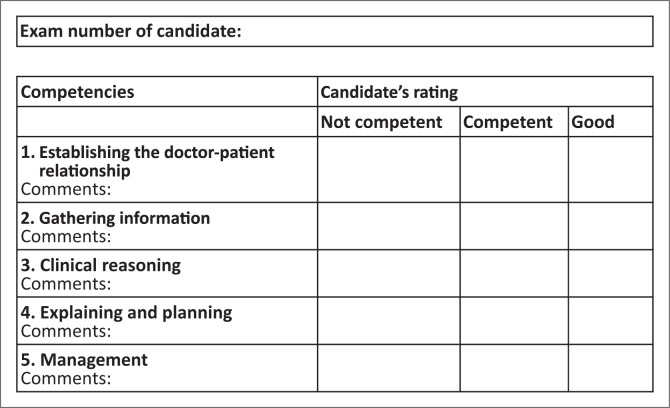
Marking template for consultation station.

No marks are allocated. In the mark sheet (Figure 2), tick off one of the three responses for each of the competencies listed. Make sure you are clear on what the criteria are for judging a candidate’s competence in each area.

Please switch off your cellphone.

Please *do not* prompt the student.

Please ensure that the station remains tidy and is reset between candidates.

### Guidance for assessors

Competency is defined as***the ability to complete a task in a safe and effective manner.*** The***examiner as an expert uses his or her judgement to categorise the candidate’s performance*.** Examiners must be well versed with the case, and the content matter.

**Establishes doctor-patient relationship:**

- The ‘competent’ candidate is respectful, listens carefully to the patient narrative, and elicits emotional cues. The ‘good’ candidate engages empathically with the patient’s suffering, facilitates the patient narrative, and responds compassionately.

**Gathering information:**

**FIGURE 3:**
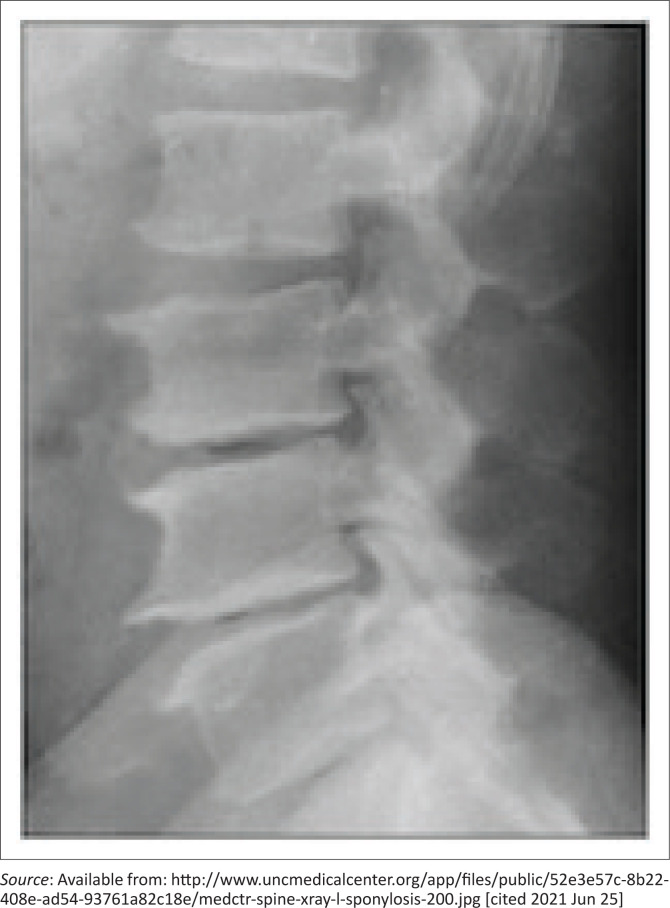
X-ray image for objectively structured clinical examination station.

- Key findings on history: pain started about a year ago, progressive worsening, starts in lower back and radiates to foot, worse when sitting for some time, no perineal or bladder impairments, impacts on function as a truck driver, breadwinner.
- Key findings from examination: increased BMI (38 kg/m^2^), limited straight leg raise on left, decreased ankle jerks on affected side, and left-sided calf muscle wasting.
- Key findings from x-ray: multi-level degenerative changes in lumbar spine (Figure 3).
- The competent candidate identifies sufficient information to develop a working diagnosis of lumbar radiculopathy and some aspects of the psychosocial dimensions/impact of the problem. The good candidate *elicits sufficient information to* make a comprehensive three stage assessment, with clear identification of ongoing risk factors.

**Clinical reasoning:**

- The competent candidate makes a working diagnosis that includes key elements (L5-S1 radiculopathy + high BMI + occupational impact). The good candidate develops a comprehensive three stage assessment, and identifies opportunities for health promotion.

**Explaining and planning:**

- The competent candidate uses non-technical language to explain the diagnosis and management plan. The good candidate shares information and actively elicits questions, provides options for intervention and co-creates a plan that is acceptable and feasible to the patient.

**Management:**

- The competent candidate has a management plan that will adequately address the key problem, incorporating pharmacological and non-pharma modalities, including the need for surgical referral. The good candidate has a comprehensive plan that includes pain management, proactively addresses ongoing risks and health promotion, within a multidisciplinary framework.

### Role play – Instructions for actors

**Patient:**

You are a 59-year-old man/woman.

**Appearance:**

Neatly dressed – casual clothes.

**Opening statement:**

‘Doctor, please help me … I have this problem which is making my life very difficult!’

Openly share:

- Started on its own about 1 year ago – no accident or injury that you remember.
- Getting worse, to the point now that it is affecting your sleep.
- You have tried ‘everything’ that you could find, but nothing seems to be helping.

Respond only if asked:

- Pain started originally in lower back, but for the last few months, also pain in back of thigh, calf and foot.
- Used Panado, Brufen, Voltaren tablets, and sometimes got Stilpane from neighbour.
- Better when you stand or walk around but can only sit for about 20 min before the pain is bad.
- No problems with bladder or bowel control, and no pins and needles around your anus or private parts.
- Habits:
  ■ Drink alcohol occasionally – special occasions only.
  ■ Stopped smoking 15 years ago.
  ■ You realise that you are overweight, but accept it – not young anymore, so not really worried about weight or fitness – work is enough exercise.
  ■ Not sleeping well, because pain is really bad.
- Work:
  ■ Drive a delivery truck for a furniture company – you don’t carry the furniture – key worry: will I be able to continue working?
  ■ Only person working in the family – two children are still studying at college, and spouse not working – ***key worry: I am the breadwinner, so can’t stop working.***
- Family:
  ■ Married, two children – home life is nice and peaceful.

### Clinical findings: provided to candidate

All vitals are normal.

BMI = 36 kg/m^2^. Waist circumference: 109 cm.

Systematic examination:

- Cardiovascular: No abnormalities.
- Respiratory: No abnormalities.
- Abdominal: No abnormalities.
- Musculoskeletal/Neurological:
  ■ Tenderness in lumbar paraspinal area – vague.
  ■ Decrease all range of movement: lower back – limited by pain.
  ■ Decreased straight leg raise on left – able to reach 20°, limited by pain in posterior thigh and back. Normal on right.
  ■ Calf on left – circumference shorter by 9 cm versus right.
  ■ Reflexes: Knee: normal bilaterally; Ankle: normal on right, decreased on left.
  ■ Power: Hip flexion: normal bilaterally; Knee flexion: normal bilaterally; Ankle flexion: 3/5 power on left.

### Further reading:

- Kies B, Mash B. How to perform a brief, appropriate neurological examination. In: Mash B, Blitz J, editors. SA family practice manual. 3rd ed. Cape Town: Van Schaik; 2015, p. 79–81.
- Mash B. How to examine and assess low back pain. In: Mash B, Blitz J, editors. SA family practice manual. 3rd ed. Cape Town: Van Schaik; 2015, p. 347–350.
- Ras T. Chronic non-cancer pain management in primary care. S Afr Fam Pract. 2020;62(1):a5203. https://doi.org/10.4102/safp.v62i1.5203

## Data Availability

Data sharing is not applicable to this article as no new data were created or analysed in this study.

